# Gene expression changes with age in skin, adipose tissue, blood and brain

**DOI:** 10.1186/gb-2013-14-7-r75

**Published:** 2013-07-26

**Authors:** Daniel Glass, Ana Viñuela, Matthew N Davies, Adaikalavan Ramasamy, Leopold Parts, David Knowles, Andrew A Brown, Åsa K Hedman, Kerrin S Small, Alfonso Buil, Elin Grundberg, Alexandra C Nica, Paola Di Meglio, Frank O Nestle, Mina Ryten, the UK Brain Expression consortium, the MuTHER consortium, Richard Durbin, Mark I McCarthy, Panagiotis Deloukas, Emmanouil T Dermitzakis, Michael E Weale, Veronique Bataille, Tim D Spector

**Affiliations:** 1 Department of Twin Research and Genetic Epidemiology, King's College London, St Thomas' Campus, Westminster Bridge Road, London SE1 7EH, UK; 2 North West London Hospitals NHS Trust, Northwick Park Hospital, Watford Road, Harrow HA1 3UJ, UK; 3 Department of Medical ƒ Molecular Genetics, King's College London, Guy's Hospital, Great Maze Pond, London SE1 9RT, UK; 4 Wellcome Trust Sanger Institute, HinxtonCB10 1SA,UK; 5 Stanford University, 450 Serra MallStanford, CA 94305, USA; 6 Wellcome Trust Centre for Human Genetics, University of Oxford, Roosevelt Drive, Oxford OX3 7BN, UK; 7 Department of Genetic Medicine and Development, University of Geneva Medical School, 1 Rue Michel-Servet (CMU office 9088), Geneva 1211, Switzerland; 8 St. John's Institute of Dermatology, King's College London, Guy's Hospital, Great Maze Pond, London SE1 9RT, UK; 9 Oxford Centre for Diabetes, Endocrinology ƒ Metabolism, University of Oxford, Churchill Hospital, Oxford, Headington OX3 7LJ,UK

**Keywords:** Aging, gene expression, skin, adipose, brain, microarrays

## Abstract

**Background:**

Previous studies have demonstrated that gene expression levels change with age. These changes are hypothesized to influence the aging rate of an individual. We analyzed gene expression changes with age in abdominal skin, subcutaneous adipose tissue and lymphoblastoid cell lines in 856 female twins in the age range of 39-85 years. Additionally, we investigated genotypic variants involved in genotype-by-age interactions to understand how the genomic regulation of gene expression alters with age.

**Results:**

Using a linear mixed model, differential expression with age was identified in 1,672 genes in skin and 188 genes in adipose tissue. Only two genes expressed in lymphoblastoid cell lines showed significant changes with age. Genes significantly regulated by age were compared with expression profiles in 10 brain regions from 100 postmortem brains aged 16 to 83 years. We identified only one age-related gene common to the three tissues. There were 12 genes that showed differential expression with age in both skin and brain tissue and three common to adipose and brain tissues.

**Conclusions:**

Skin showed the most age-related gene expression changes of all the tissues investigated, with many of the genes being previously implicated in fatty acid metabolism, mitochondrial activity, cancer and splicing. A significant proportion of age-related changes in gene expression appear to be tissue-specific with only a few genes sharing an age effect in expression across tissues. More research is needed to improve our understanding of the genetic influences on aging and the relationship with age-related diseases.

## Background

Aging has been described as a progressive decline in the ability to withstand stress, damage, and disease resulting in degeneration [1,2]. Age is also a major risk factor in the development of many diseases, although the relationship between the aging process and the etiology of age-related diseases is not fully understood. Previous gene expression studies of aging have primarily concentrated on model organisms [3] or have been confined to specific aging-associated disorders such as progeria syndromes[4]. A study of postmortem human brain tissue from 30 individuals aged 26 to 106 years [5] showed that approximately 4% of approximately 11,000 genes analyzed show a significant age-related expression change (1.5-fold or more) in individuals aged >40 years. These genes were reported to play central roles in synaptic plasticity, vesicular transport, and mitochondrial function. Another study [6]examined gene expression changes with age in healthy renal tissue removed at nephrectomy from 74 patients ranging in age from 27 to 92 years old; identifying 985 genes differentially expressed with age. More recently, a meta-analysis of age-related gene expression profiles combined multiple disparate gene expression studies in an attempt to identify common signatures of aging across both tissue and species [7]. However, to date, studies published using human tissuehave all been carried out on a limited number of samples, making them underpowered for the detection of normal age-related expression differences.

**Figure 1:**
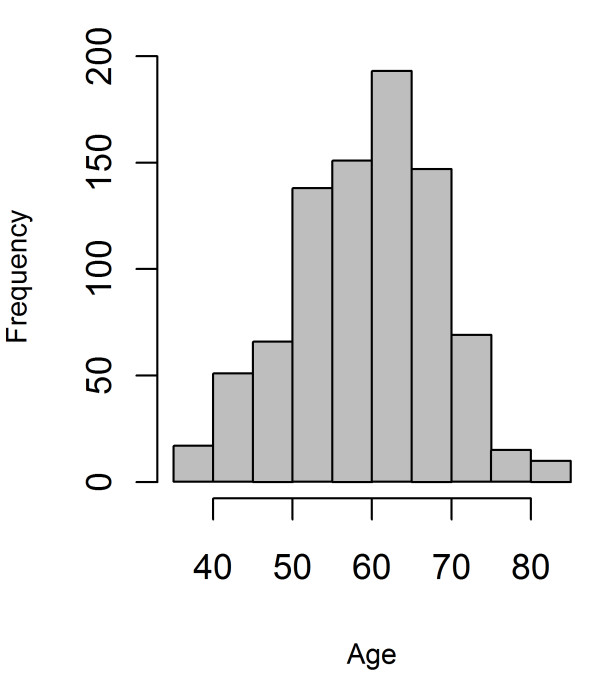
**Histogram showing the age distribution of the individuals in the study**.

The aims of this study were to determine which genes and pathways show differential expression with age in multiple tissues and to understand how the genomic regulation of gene expression alters with age. Age effect on gene expression was explored by examining expression profiles in skin, adipose tissue, and lymphoblastoid cell lines (LCLs) from 856 female twins agedfrom 39 to 85 yearsold (Figure 1)from the Multiple Tissue Human Expression Resource (MuTHER study) [8].Genes significantly affected by age in skin and adipose tissues were followed upin 932 postmortembrain samples (representing from 10 brain regions) from 100 individuals;provided by the UK Brain Expression Consortium[2]. In addition, the influence of genetic variants on gene expression in aging individuals was explored by examining significant eQTL from the MuTHER dataset [9], for a genotype-by-age interaction.

## Results

### Age-related gene expression

**Figure 2:**
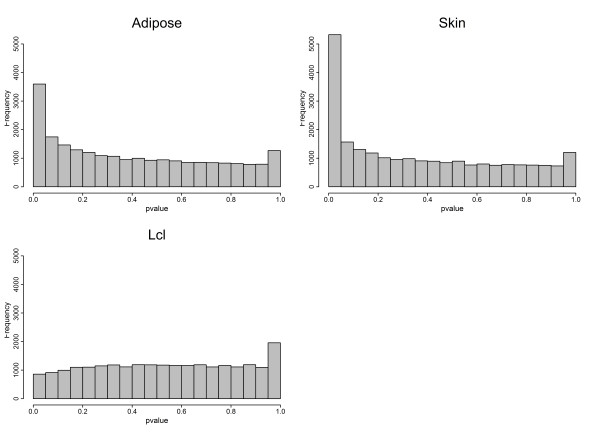
**Venn diagram showing the overlap between significant age-affected genes in skin and adipose tissues (Benjamini-Hochberg corrected *P*value < 0**.01).

**Figure 3:**
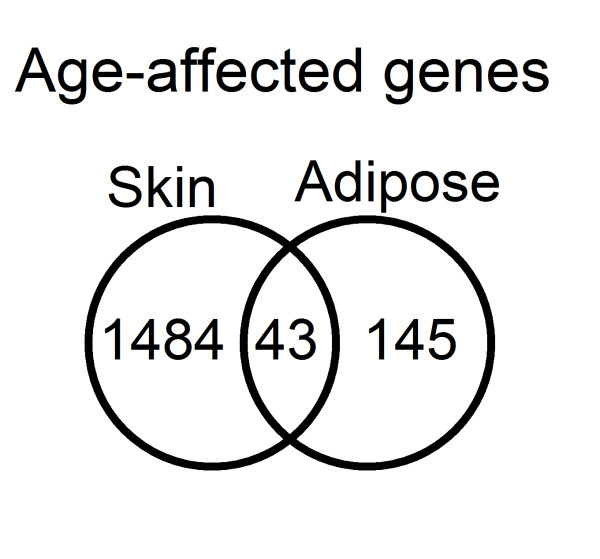
***P*values distribution of age-affected genes in adipose, skin, and LCL tissues**.

Expression profiles were determined from 856 individuals aged between 39 and 85 years from skin, adipose tissue,and LCL samples using the Illumina Human HT-12 V3 Bead chip.All the volunteers were female twins(336 MZ and 520 DZ) recruited from the TwinsUK Adult twin registry [10] aged 39 to 85 years old, with an average age for the cohort of 59.3 years (Figure 1 and Additional file 1, TableS 1).A linear mixed model identified 1,672 genes as differentially expressed with chronological age in skin, 188 in adipose tissue, and two in LCLs (Benjamini-Hochberg corrected *P*value < 0.01)(Figures 2 and 3, and Additional files 2 and 3, Tables S2 and S3). Of those genes, 43 were found in both skin and adipose tissue. The direction of the age effect on gene expression was similar in each tissue for most of the genes; 48.6% of the genes had lower levels of expression with age in skin compared to50.8% in adipose tissue. Six genes exhibited age effects in opposite directions in different tissues. Two genes, the chromosome 5 open reading frame 4 (*C5orf4*) and the spondin 1 (*SPON1*) showed lower expression with age in skin but higher in adipose tissue. Conversely, four genes showed higher expression levels with age in skin but lower in adipose tissue. Those genes were carbonic anhydrase XII (*CA12*), solute carrier family 47, member 1 (*SLC47A1*), Rho GTPase activating protein 33 (*ARHGAP33 *or *SNX26*), and B-cell CLL/lymphoma 6 (*BCL6*).

**Table 1:** Significantly age-affected genes (in grey) from brain transcription profiles in brain regions with significant values.

Affy transcript ID	Affy Probe ID	Illumina Probe	Symbol	CRBL	HIPP	PUTM	TCTX	WHMT	Skin	Adipose
t2378077	NA	ILMN_1811370	HSD11B1	2.89E-08	2.80E-01	8.90E-01	2.07E-01	3.58E-01	2.33E-09	7.40E-01

t2378077	205404_at	ILMN_2389501	HSD11B1	2.89E-08	2.80E-01	8.90E-01	2.07E-01	3.58E-01	3.53E-11	4.31E-01

t2378077	NA	ILMN_2389506	HSD11B1	2.89E-08	2.80E-01	8.90E-01	2.07E-01	3.58E-01	2.82E-09	7.11E-01

t3219885	203997_at	ILMN_1717294	PTPN3	1.52E-05	6.45E-01	8.28E-01	2.09E-01	8.81E-01	5.08E-04	1.88E-01

t3883207	203650_at	ILMN_1717262	PROCR	3.85E-05	5.78E-01	9.16E-01	8.73E-01	6.27E-01	5.83E-03	2.09E-02

t3168066	205199_at	ILMN_1725139	CA9	6.79E-03	8.11E-01	9.75E-01	9.70E-01	9.92E-01	9.75E-03	5.86E-01

t3349719	NA	ILMN_1750496	ZBTB16	3.13E-04	3.17E-01	8.28E-01	9.38E-01	5.35E-01	1.00E+00	7.71E-03

t2753732	222738_at	ILMN_2207170	WWC2	3.62E-04	9.49E-01	9.28E-01	7.53E-01	7.32E-01	1.84E-01	1.91E-03

t2316605	206080_at	ILMN_2061565	PLCH2	3.05E-01	6.94E-01	8.85E-01	7.00E-03	4.43E-01	1.27E-05	8.98E-01

t3690154	218888_s_at	ILMN_1760849	NETO2	7.64E-01	1.94E-01	8.72E-01	9.43E-03	5.77E-01	3.39E-04	9.21E-01

t3374934	230550_at	ILMN_1721035	MS4A6A	3.05E-01	5.89E-05	1.38E-04	1.39E-01	9.35E-01	2.56E-05	9.33E-01

t3374934	219666_at	ILMN_1797731	MS4A6A	3.05E-01	5.89E-05	1.38E-04	1.39E-01	9.35E-01	9.10E-04	8.87E-01

t3692999	NA	ILMN_1715401	MT1G	3.49E-01	2.10E-01	1.83E-03	3.09E-01	2.16E-01	3.57E-04	8.42E-01

t3703885	201195_s_at	ILMN_1720373	SLC7A5	5.80E-02	5.09E-01	8.28E-01	7.54E-01	3.50E-03	1.54E-07	8.96E-01

t2830465	219728_at	ILMN_1656395	MYOT	6.65E-01	5.50E-02	8.28E-01	2.77E-01	4.04E-03	4.08E-03	9.30E-01

t2924492	219743_at	ILMN_1682034	HEY2	7.26E-01	1.56E-01	7.31E-01	9.63E-02	2.31E-03	2.99E-04	5.77E-01

t2478269	NA	ILMN_1678403	TMEM178	8.91E-01	6.45E-01	9.20E-01	2.20E-01	2.31E-03	9.81E-03	6.41E-05

t2478269	229302_at	ILMN_2104295	TMEM178	8.91E-01	6.45E-01	9.20E-01	2.20E-01	2.31E-03	2.26E-05	7.67E-08

Benjamini-Hochberg corrected *P*values for adipose and skin genes are also shown. Abbreviations:CRBL =Cerebellum, HIPP= Hippocampus, PUMT= Putamen, TCTX= Temporal cortex, WHMT= Intralobular white matter.

GO processes and gene expression enrichment for functional classification were investigated using DAVID [11]. In skin the enrichment included terms related to epidermal development, keratinization, epithelial cell differentiation, extracellular matrix organization, intermediate filament cytoskeleton, collagen fibril organization, and fatty acid metabolic processes(Benjamini-Hochberg corrected *P*value < 0.05).The enrichment analysis of those genes in skin that significantly increase expression with age identified GO terms relating to alternative splicing and RNA processing as well as cell and organelle structure-related cellular components. Genes that have lower expression with age in skin were enriched for gene ontology terms related to metabolism, biosynthetic processes, and mitochondrial function. In adipose tissue, enrichment analysis only revealed one significant GO term: extracellular structure organization. Separate analysis of genes in fat with either increased or decreased expression did not identify any enriched term. Enrichment analysis in DAVID of the genes in common between tissues (43 genes, Figure 1) did not identify any significant GO terms but were enriched for genes with a tissue-specific expression in the adrenal cortex, as defined by the Genomics Institute of the Novartis Research Foundation [12]. The adrenal cortex forms an active part of the hypothalamic pituitary adrenal (HPA) axis, implicated in the aging process as being the central method by which the body responds to stress, a response which may transcend tissue-specific aging[13].

We also investigated whether known aging-related genes from The Human Ageing Genomic Resources (HAGR)[7,14]were included in the genes found to be differentially expressed in skin and adipose tissues in our dataset. Within HAGR, GenAge is a database of 288 genes potentially associated with human aging, 25 of which were found to be differentially expressed with age in skin and three in adipose tissue in our study(Additional file 4, Table S4).

Many of the age-altered expression genes in skin and adipose tissue belong to cancer-related pathways like p53, Wnt, or Notch, or have been previously implicated in many cancer types. In skin, some of the genes belong to the p53 pathway like p21 (*CDKN1A*), tripeptidyl peptidase 1 (*TPP1*), and the tumor protein p53 regulated apoptosis inducing protein 1 (*TP53AIP1*). Also, differentially expressed genes with age in skin belong to the Wnt and the NOTCH pathways like *WNT4*, *WNT3 *or the SMAD family member 3 (*SMAD3*), the jagged 2 (*JAG2*) or the nuclear receptor corepressor 2 (*NCOR2*). In adipose tissue, other genes differentially expressed with age were also related to p53 or cancer and include the vascular endothelial growth factor C (*VEGFC*), the ret proto-oncogene (*RET*), the zinc finger and BTB domain containing 16 (*ZBTB16*) or notch 3 (*NOTCH3*).

### LCLs expression showed little significant age effect

LCLs are derived from lymphoblastoid cell lines infected and immortalized by Epstein-Barr virus (EBV). Analysis of age influence on gene expression in 777 LCLs samples identified only two genes differentially expressed with age (Benjamini-Hochbergcorrected *P*value < 0.01), a gene coding for aspartoacylase (*ASPA*) and the OUT domain containing 7A (*OTUD7A*) (Additional file 5, Table S5). Previous studies analyzing age influence in LCLs expression profiles found also very little influence of chronological age [15]. Although, Joehanes *et al. *[15] attributed their results to a small sample size, the larger sample size here analyzed added further weight to the hypothesis that there is a lack of detectable chronological age effect on gene expression in transformed lymphocytes. To further investigate the lack of age-related expression differences, expression profiles from 92 fresh lymphocytes obtained from a subset of the individuals were analyzed. No gene showed a significant age-related effect (Benjamini-Hochbergcorrected *P*value < 0.01)(Additional file 6, Table S6). However, based on the *P*value distribution and the effect sizes detected in the smaller number of fresh lymphocytes compared to transformed lymphocytes (LCLs), a small age effect of fresh lymphocytes cannot be ruled out (Additional files 7, 8, 9, Figures S1-S3).

### Age-affected genes in brain

To explore common expression signatures of aging in other tissues, expression data from the Edinburgh brain bank were interrogated [16]. The brain expression dataset used 932brain samples obtained from 10 different brain regions following sudden death in 100 individuals aged 16 to 83 years. This dataset was examined for transcripts that were differentially expressed with age. Of 1,860 skin or adipose tissue transcripts with a significant age effect described above, 14 brain genes in five of the 10 available brain regions were found to have a significant age-altered expression (Benjamini-Hochberg corrected *P*value <0.01, Additional file 10, Table S7). Of those 14 genes (Table 1), one was common to all the three tissues (*TMRM178*);two more genes were in common between adipose and brain only, and 12 more genes between skin and brain. The two genes common between adipose tissue and brain tissue (cerebellum) were a zinc finger transcription factor coding gene (*ZBTB16*) and the WW and C2 domain containing two genes (*WWC2*). From the 12genes common to skin and brain, four were differentially expressed with age in cerebellum (*PROCR, HSD11B1, PTPN3*,and *CA9)*, two in temporal cortex (*PLCH2 *and *NETO2*),one in putamen (*MT1G*),four in intralobular white matter (*SLC7A5*, *MYOT*,*HEY2 *and *TMEM178*),and one in hippocampus and putamen (*MS4A6A*).

Many of the age-altered expression genes in brain have been also associated with cancer and other aging-related diseases. *ZBTB16 *(*PLZF*) has previously been implicated in acute myeloid leukemia development via protein fusion with the retinoic acid receptor alpha gene product and interaction with p53 [17]. The protein C receptor *(PROCR) *belongs to the blood coagulation pathway and has been linked both to aging-associated diseases (like cardiovascular diseases) and cancer [18]. The carbonic anhydrase IX (*CA9*) is a transmembrane enzyme implicated in development that has also been associated with cancer in multiple organs including the brain [19]. Additionally, *CA9 *mediated changes in *SIRT1*, a sirtuin involved in cellular differentiation and stress response whose activity reduces p53 mediated apoptosis and FOXO-induced apoptosis [20-22]. *SLC7A5*is an amino acid transporter light chain, also known as *LAT1*, that has been established as a biomarker for the development of cancer [23].Another age-related disease gene in brain was*MS4A6*, a membrane-spanning four-domains gene which has been associated with Alzheimer's disease [24], a known aging-related disease. Finally *TMEM178*, the only gene found to be affected by age in skin, adipose tissue, and brain (intralobular white matter) is a transmembrane protein whose function is unknown but has been previously linked to age-related changes in gene expression of murine lung tissue [25].

### Genotype-by-age interactions (eQTL analysis)

**Figure 4:**
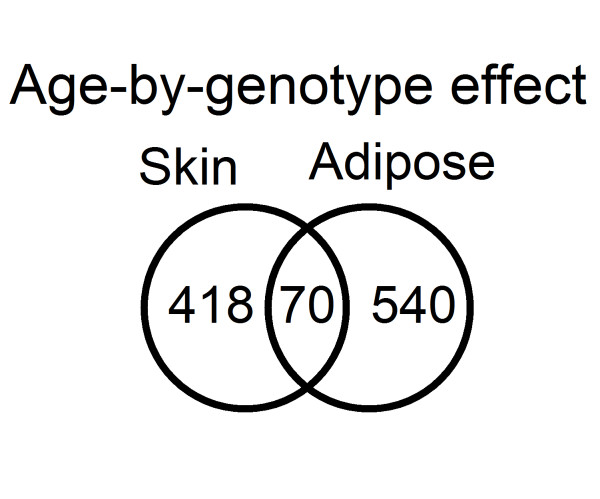
**Venn diagrams showing the overlap in skin and adipose tissue for GxA interacting genes**.

A common approach to identify factors controlling differential gene expression is to incorporate genomic data to derive a set of expression QTL (eQTL)[26]. Specific gene allele differences may cause variation in gene expression with age and may be detectable by eQTL analysis. Since eQTL identify SNPs that influence the expression of a gene, GxA interactions identify a difference in the SNPs influence on gene expression due to an interaction with age. It suggests that the effect of the SNP on gene expression is not constant throughout the life span of the individual and that age plays a relevant role in the effect that the genetic variant has on gene expression.Therefore, we included an age term in an eQTL analysis to investigate possible genotype-by-age (GxA) interactions that explain differences in gene expression with age among individuals. By determining which eQTL show an age interaction, we have the potential to explain some of the genetic control in age-related gene expression changes and hence differences in aging rates between individuals. We limited our analysis to 3,529 SNPs in adipose tissue and 2,796 SNPs in skin that had been identified as significant eQTL by the MuTHER study[27]. A full linear mixed model with a GxA interaction term was compared with a model without an interaction term and evaluated using the Akaike Information Criterion (AIC). Interaction effects were identified with a better model fit for 610 eQTL in adipose tissue and for 488 eQTL in skin. Of these, 70 eQTL were common to both tissues (Figure 4). These were explored by the UK Brain Expression Consortium dataset but no significant interactions (*P*value<1e-5) were identified.

Genes with an GxA interaction in both skin and adipose tissue included a member of the B-cell lymphoma family (*BCL7C*)as well as the *BRCA1 *associated RING domain 1 gene (*BARD1*) and the YEATS domain containing 4 (*YEATS4*, also known as *GAS41*), both of which are p53 related genes previously associated with cancer. Among the genes in skin with a significant GxA interaction we found 31 genes associated with cancer, including the Telomerase reverse transcriptase (*TERT*), which is also involved in aging via its effect on telomere length [28]. Others include the fibroblast growth factor receptor 4 (*FGFR4*), the interferon gamma receptor 2 (*IFNFR2*), and the heat shock 70kDa protein 1A (*HSPA1A*) (Additional files 11 and 12, Tables S8 and S9). In adipose tissue, we found not only a number of known cancer-related genes, like *SOD2 *or *AMACR*, but also diabetes- and obesity-related genes, like *MTHFR *or *ADIPOR2*.

Genetic markers of diseases identified by GWAS have been often associated with gene expression changes via eQTL studies. Integration of cis-eQTL data from the MuTHER study and published disease loci has previously identified eQTL associated with diseases [27]. In skin, three SNPs with a GxA interaction were associated with smoking behavior, electrocardiographic traits, and tooth development. Twenty-five SNPs with a GxA interaction identified in adipose tissue (Table 2) were associated with age-related traits and diseases like Parkinson's (rs10516849, rs708726) or cancer (rs402710, rs6715570, rs12912744). Interestingly, one of the disease-associated variants (rs402710) underlying a GxA cis-eQTL in the cleft lip and palate associated transmembrane protein 1 gene (*CLPTM1L*) has been previously associated to lung cancer susceptibility and is located in a LD region that includes *TERT*[29].

**Table 2:** List of SNPs with an age-by-genotype interaction effect associated to GWAS hits.

eQTL	Gene	Trait
**Adipose**		

rs10516849	MMRN1	Parkinson's disease

rs10870077	<NA>	Ulcerative colitis

rs12006032	PRKACG	Chronic kidney disease

rs12523750	MRS2	Radiation response

rs12912744	SERF2	Lung cancer

rs17253722	SHRM	Serum magnesium levels

rs17253722	SHRM	Chronic kidney disease;renal function and chronic kidney disease

rs17253722	SHRM	Eosinophilic esophagitis (pediatric)

rs17671591	C5orf37	LDL cholesterol

rs17671591	C5orf37	Quantitative traits

rs402710	CLPTM1L	Lung cancer

rs402710	CLPTM1L	Bladder cancer;pancreatic cancer

rs531676	CRTAC1	Metabolic syndrome

rs592229	AGPAT1	Menopause (age at onset)

rs592229	AGPAT1	Height

rs6143035	CABLES2	Colorectal cancer

rs6715570	BARD1	Neuroblastoma (high-risk)

rs708726	RAB7L1	Parkinson's disease

rs8049897	DBNDD1	Blond *vs*. brown hair color;freckles;red*vs*. non-red hair color;skin sensitivity to sun

rs8049897	DBNDD1	Black *vs*. red hair color;melanoma

rs9263871	HCG27	Protein quantitative trait loci

rs9263871	HCG27	Vitiligo

rs9263871	HCG27	CD4:CD8 lymphocyte ratio

rs9926577	CNOT1	QT interval

**Skin**		

rs2305797	RAB4B	Smoking behavior

rs1049337	CAV1	Electrocardiographic traits;PR interval

rs1042815	HOXB2	Primary tooth development (number of teeth)

## Discussion

Aging studies from model organisms such as yeast, worms, and flies have repeatedly shown that changes in the expression of certain genes have an effect upon longevity. Although similar aging processes are likely to operate across multiple species [30], it has been much more difficult to identify longevity candidate genes in human studies [30]. A key question in human aging is to what extent a signature of aging may be detectable across tissues. Until now there has been a lack of large transcriptional profiles from the same human individuals in multiple tissues. The MuTHER study provides insight into the human aging process by interrogating the largest multiple human tissue gene expression resource to identify genes in which expression was affected by chronological age. The analysis of the skin and adipose tissues samples identified several hundred genes responsive to changes in chronological age. However, the 43 shared genes in skin and adipose tissue showed a single common identifiable pathway related to the stress response. From over 1,800 transcripts that have altered expression with age in skin and adipose tissues, 14 also had age-related differential expression in brain. The limited overlap in these two experiments may partly reflect the smaller sample size of the brain expression dataset, the differences in age range between the studies (16 to 83 years for brain samples; 39 to 85 years for MUTHER samples), or the inclusion of males in the brain samples. But it may also imply, as other studies have suggested, that the effects of age on gene transcription are tissue specific[6,31,32]. This hypothesis was supported by the comparison with known related aging genes from the GenAge database, which identified an overlap for a small number of aging-related genes with our data. The GenAge database was the result of a meta-analysis using age-related expression profiles from human brain, kidney, and skeletal muscle, and several expression profiles from mouse and rat; no adipose tissue or skin samples were included (Additional file, Table 1 in [7]). The limited overlap between these datasets supports the idea that molecular signatures of aging reflect predominantly a tissue-specific transcriptional response.

The lack of age-related genes in transformed LCLs, suggest that the transformation to immortalize a cell line may mask or even remove the age-related signatures in gene expression. The transformation of primary B lymphocytes into LCLs requires infection by the Epstein-Barr virus which has the effect of disrupting the p53 signaling pathway in order to induce growth and survival [33]. Joehanes *et al.*[15] identified only five genes with age-associated expression in LCLs, including p53 itself (*TP53*). Although the authors attribute the lack of age-affected genes to their small sample size (*n*=50) and narrow age range, our analysis with a much larger sample size found even fewer age-related changes, suggesting a lack of detectable aging signature in LCLs. The analysis in the subset of fresh lymphocytes suggested an age influence in fresh lymphocytes may potentially be detectable with a larger sample size.

Similarly to age-affected genes, genes with a significant GxA interaction are likely to be relevant for the general aging process. More than 300 GxA interactions have previously been identified in the model organism *C. elegans*[34,35]and more than 600 have been documented in human blood and kidney [36,37]. By focusing on SNPs with a statistically significant effect on expression (eQTL) from the MuTHER study, we were able to identify more than 1,000 genes for which a GxA interaction better explained the SNP effect in expression in skin and adipose tissue. Fewer than 10% of GxA interactions were observed in both tissues, suggesting that these changes were also tissue-specific. Model organism studies have observed that gene expression can influence the aging rate of an individual but also the aging rate of different tissues[38]. A feedback process may be involved here, where the consequences of the aging process may affect the expression of multiple genes like the expression and regulation of gene expression may affect the aging process. However, it is impossible to determine whether the gene expression changes observed were result or causative of the aging process. The GxA interactions identified the interface between tissue-specific gene expression regulation and tissue-specific aging. But resolving the potential feedback mechanism would require deeper knowledge of the regulatory process of gene expression and its relationship with the aging process. Our results, however, provide a starting point for future work in this direction.

Among the 70 genes associated with GxA common to both tissues, we identified cancer-related genes, such as *BARD1 *and *BCL7C*.The *BARD1/BRCA1 *interaction is disrupted by tumorigenic amino acid substitutions in *BRCA1*, implying that the formation of a stable complex between these proteins may be an essential aspect of *BRCA1 *tumor suppression. *BARD1 *may also be the target of oncogenic mutations in breast or ovarian cancer and is also important for DNA repair. *CLPTM1L *gene, which has been implicated in susceptibility to lung cancer, had a GxA effect in both skin and fat. A recent study has shown that the *TERT*-*CLPTM1L *locus is also associated with melanoma risk [39]. This suggests that the associations of genetic variants to certain diseases, in particular the onset of cancer, are modified by chronological age-related effects. Other studies have found associations between genes expression changes with age and the development of cancer [40,41] and while age remains the strongest risk factor for developing the vast majority of cancers, the actual relationship between the aging process and the development of cancer is complex and far from fully understood[42,43]. We provide a description of genes affected by age in multiple tissues. Using the limited knowledge on gene functionality available on the databases, many of those genes have been associated to cancer, in some way. However, we do not talk in terms of enrichment for 'cancer genes' as we are not able to define them accurately enough to generate a set of genes to test in a statistical analysis. Are oncogenes considered the only cancer genes? What about metastasis and tumour suppressor genes? What about other genes? After all, genes involved in authophagy, cell migration, and metabolism in general are also involved in cancer, with immune-system-related genes also being relevant for this process. The distinction is still unclear making the definition of a list of 'cancer' genes difficult. Since epidemiological studies identify age as a strong and consistent risk factor for cancer, it is well accepted that both processes are related, but the actual mechanism behind is still unknown. Our study supports the idea that gene expression changes with age might be linked to the association between aging-related diseases and senescence or lack of it, as both processes share many genes. Recently this relationship has been exploited when using pro-senescence therapies for cancer treatment [44]. Although secondary effects of these therapies are still under investigation, the positive results obtained in animal models suggest that genes which expression changes with age may be relevant to understand the link between cancer and aging.

Regarding cancer and aging, Serrano and Blasco (2007) suggested that an equilibrium between mechanisms diminishing cellular damage and mechanisms preventing excessive cellular proliferation is required between both processes[43]. The authors argue that the p53 pathway may be seen as an anti-aging mechanism as it is a key defense mechanism against cellular damage protecting from both aging and cancer. One effect of aging at the cellular level is reduced telomerase activity and progressive shorter telomeres in somatic cells [45]. Shortened telomeres are highly recombinogenic, leading to a genome-susceptible cancer development[46,47]. Genomic instability driven by dysfunctional telomeres is also associated with the transition from benign to malignant tumors[48]. Conversely, telomere dysfunction also acts to induce the p53 gene to suppress tumor development by initiating cell-cycle arrest, cellular senescence or, apoptosis. Our analysis has identified several genes involved in the regulation and activity of the p53 pathway as being affected by age. In skin, the telomerase reverse transcriptase (*TERT*) showed an age-related expression in association with a genetic variant (rs10866530). In addition p21, a gene directly regulated by p53 and also involved in telomere-driven aging, was shown to be differentially expressed with age [49]. In brain, the*ZBTB16, CA9*,and *HEY2*, genes associated to the p53 pathway directly or via *SIRT1*, all showed age-related expression. The activity of p53 has been shown to enhance the transcription of inhibitors of the insulin receptor pathway, preventing cell growth and division after stress signaling[50,51] and many genes from the insulin signaling pathway have been extensively associated with longevity in multiple studies and organisms. Our results suggest that the link between aging and cancer is evident in multiple tissues through differential expression of genes with age.

## Conclusion

This study examines for the first time changes in gene expression with chronological age in multiple normal human tissues from the same individuals. While a significant proportion of age-related changes in gene expression appear to be tissue-specific, a few common genes were affected by age in a diverse range of tissues. Many of these genes are important in cell division regulation, senescence and apoptosis, processes prone to dysregulation, and potential oncogenesis with advancing age. Tissues comparison suggested that a significant proportion of age-related changes in gene expression are tissue-specific. This raises the question of to what extent age-related changes in gene expression are due to tissue aging rate differences or to tissue-specific gene expression regulation. Further interrogation of this aging-related expression and eQTL resource has the potential to unravel the complex interactions betwe enaging and gene expression regulation, as well as the link with age-related diseases. However, this may reveal that the two processes are so inextricably linked that it is difficult to consider them independently.

## Materials and methods

### Sample collection

The study included 856 Caucasian female individuals (336 MZ and 520 DZ twins, respectively) recruited from the TwinsUK Adult twin registry [39]. The age at inclusion ranged from 39 to 85 years with a mean age of 59 years. Punch biopsies (8mm) were taken from relatively photo-protected infra-umbilical skin. Subcutaneous adipose tissue was carefully dissected from the biopsy site using forceps and scalpel, weighed, and immediately stored in liquid nitrogen. Similarly, the remaining skin tissue was weighed and stored in liquid nitrogen. Peripheral blood samples were collected, and LCLs were generated through EBV-mediated transformation of the B-lymphocyte component by the European Collection of Cell Cultures agency. The data presented here are part of the MuTHER project (Multiple Tissue Human Expression Resource)[52], Nica *et al.*[9],and Grundberg*et al. *[8].The project was approved by the local ethics committee of all institutions involved. All the samples were collected after obtaining written and signed informed consent, in accordance with the Helsinki Declaration.

### RNA extraction

RNA was extracted from homogenized tissue samples (adipose and skin) and lysed cells (LCL) using TRIzol Reagent (Invitrogen) according to protocol provided by the manufacturer. RNA quality was assessed with the Agilent 2100 BioAnalyzer (Agilent technologies) and the concentrations were determined using NanoDrop ND-1000 (NanoDrop Technologies) and samples were stored in -80°C until ready to use. cDNA derived from the RNA sample was hybridized with the Illumina Human Sentrix 12 chip.

### Expression profiling

Expression profiling of skin, adipose tissue, LCLs, and fresh lymphocites, each with either two or three technical replicates, were performed using the Illumina Human HT-12 V3 BeadChips (IlluminaInc) including 48,804 probes where 200ng of total RNA was processed according to the protocol supplied by Illumina. All samples were randomized prior to array hybridization and the technical replicates were always hybridized on different beadchips. Raw data were imported to the IlluminaBeadstudio software and probes with fewer than three beads present were excluded. Log2-transformed expression signals were then normalized separately per tissue with quantile normalization of the replicates of each individual followed by quantile normalization across all individuals as previously described [9]. Post-QC expression profiles were subsequently obtained for 825 (adipose tissue and LCL), 705 (skin), and 92 (fresh lympholytes) individuals, respectively. The Illumina probe annotations were cross-checked by mapping the probe sequence to the NCBI Build 36 genome with MAQ. Only uniquely mapping probes with no mismatches and either an Ensembl or RefSeq ID were kept for analysis. Probes mapping to genes of uncertain function (LOC symbols) and those encompassing a common SNP (1000G release June 2010) were further excluded leaving 23,596 probes used in the analysis. Microarray data can be download from the ArrayExpress archive, accession no. E-TABM-1140.

### Analysis of gene expression with age

A linear mixed model was used to examine gene expression variability by age and confounding factors including as fixed effect batch and RNA concentration (only in skin samples), and as random effects family relationship and zygosity. We fitted the mixed-effects model in R[53] with the lmer function in the lme4 package [54]. The *P*values to assess significance for age effect were calculated from the Chi-square distribution with 1 degree of freedom using likelihood ratio as the test statistic. We computed *P*values adjusted for multiple testing by controlling the false discovery rate (FDR) with the Benjamini-Hochberg procedure [55]in R and using a threshold of 0.01. Enrichment analysis was carried out using the DAVID Bioinformatics Resource server with a significant level threshold of 0.05 in Benjamini-Hochberg corrected *P*values [11].

### Genotyping and imputation

Genotyping of the TwinsUK dataset (*n*= approximately 6,000) was done with a combination of Illumina arrays (HumanHap300, HumanHap610Q, 1M-Duo, and 1.2MDuo 1M).Intensity data for each of the three arrays were pooled separately (with 1M-Duo and 1.2MDuo 1M pooled together) and genotypes called with the Illuminus calling algorithm, thresholding on a maximum posterior probability of 0.95.Similar exclusion criteria were applied to each of the three datasets separately. Exclusion criteria for samples were: (i) sample call rate < 98%, (ii) heterozygosity across all SNPs ≥2 s.d. from the sample mean; (iii) evidence of non-European ancestry as assessed by PCA comparison with HapMap3 populations; and (iv) observed pairwise IBD probabilities suggestive of sample identity errors. Exclusion criteria for SNPs were: (i) Hardy-Weinberg *P*value<10-6, assessed in a set of unrelated samples; (ii) MAF<1%, assessed in a set of unrelated samples; and (iii) SNP call rate <97% (SNPs with MAF≥5%) or <99% (for 1% ≤MAF <5%).

Prior to merging the three datasets, we performed pairwise comparison among the three datasets and further excluded SNPs and samples as follows: (i) concordance at duplicate samples <1%; (ii) concordance at duplicate SNPs <1%; (iii) visual inspection of QQ plots for logistic regression applied to all pairwise dataset comparisons; (iv) Hardy-Weinberg *P*value <10-6, assessed in a set of unrelated samples; and (v) observed pairwise IBD probabilities suggestive of sample identity errors.

Imputation was performed using the IMPUTE software package (v2)26 using two reference panels, P0 (HapMap2, rel 22, combined CEU+YRI+ASN panels) and P1 (610k+, including the combined TwinsUK HumanHap610k and 1M array).Post-imputation, SNPs were filtered at a MAF >5% and IMPUTE info value of >0.8.

### The UK Brain Expression Consortium dataset

The samples from the UK Human Brain Expression Consortium [16] used in this study were provided by the MRC Sudden Death Brain and Tissue Bank in Edinburgh [56] and originated from 100 individuals (78 men and 22 women) of European descent. For each individual, up to 10 anatomical brain regions were sampled: cerebellar cortex (CRBL), frontal cortex (FCTX), hippocampus (HIPP), medulla (more specifically the inferior olivary nucleus, MEDU), occipital cortex (OCTX), putamen (PUTM), substantianigra (SNIG), temporal cortex (TCTX), thalamus (THAL), and intralobular white matter (WHMT). A detailed description of the samples used in the study, tissue processing, and dissection is provided in Trabzuni*et al*. (2011). All samples had fully informed consent for retrieval and were authorized for ethically approved scientific investigation (Research Ethics Committee number 10/H0716/3).

The tissues were profiled using the Affymetrix Human Exon 1.0 ST array (*n*=932 arrays) and subsequently preprocessed using RMA using a high confidence list of probesets (unique hybridization, gene annotation, and at least three valid probes after removal of probes containing SNPs). Exon-level expression data was corrected for sex and batch effects and summarized into transcript-level expression values using 10% trimmed mean.

Each individual was genotyped on two chips: the IlluminaInfinium Omni1-Quad BeadChip and the ImmunoChip, a custom genotyping array designed for the fine-mapping of auto-immune disorders [57,58]. Individuals suspected of being of non-European ancestry were identified using principal components projection and excluded from analysis. After standard quality controls, both genotype datasets were combined and imputed using MaCH[59,60]and minimac [61]using the 1000Genomes (March 2012). We used the resulting approximately 5.8 million SNPs with good post-imputation quality (r2 >0.50) and minor allele frequency of at least 5% in subsequent analyses.

The Illumina IDs were converted to Affymetrix transcript ID using available annotation files from the two company websites and matched to the best sequence similarity. This resulted in a match for 63.6% of the genes of interest. For the remaining genes, we matched by gene symbol. Overall, approximately 98% of the replication list was matched in the UK Brain Expression Consortium. The log-likelihood comparing a model that regressed gene expression against age with a model with the intercept only was calculated and tested under an F-distribution with 1 degree of freedom and the corresponding *P*values reported here and corrected with the Benjamini-Hochberg procedure for FDR correction.

### Genotype-by-age (GxA) interaction effect on gene expression

The genetic effect of transcripts to imputed genotypes by genome wide *cis*-eQTL mapping on more than 23,500 expression traits was previously mapped in three tissues [7]. In total, 776 adipose, 777 LCL, and 667 skin samples had expression profiles and imputed genotypes and were included in the analysis. Across all transcripts significant eQTL were 3,529 in adipose, 4,625 in LCL, and 2,796 in skin. Here we investigate the genotype-by-age (GxA) interaction contribution to gene expression variance observed in those genes. For that we question each gene and the most significant SNP (pick of the eQTL) association with a linear mixed model that included GxA interaction as contributing factor to the gene expression variance. The linear mixed model test was done using the lmer() function in the lme4 package [45] and adjusted for age, experimental batch effect, and sample processing effect in skin (fixed effects) and for family relationship and zygosity (random effects). The Akaike Information Criterion (AIC) was used as a selection method to identify the best fit explaining the origin of the variation per gene expression. Genes for which the model with a genotype-by-age interaction factor fit better the data were used in the functional analysis.

### Accession codes

Microarray data of skin, adipose tissue, LCLs can be download from the ArrayExpress archive, accession no. E-TABM-1140. Details about the MuTHER Resource can be found in the bibliography [8,52].

Microarray data of brain can be downloaded from GEO, accession no. GSE46706. Details about the UK Brain Expression Consortium and expression resource can be found in the bibliography[2,62]

### Ethics Statement

This project was approved by the ethics committee at St Thomas' Hospital London, where all the biopsies were carried out. Volunteers gave informed consent and signed an approved consent form prior to the biopsy procedure. Volunteers were supplied with an appropriate detailed information sheet regarding the research project and biopsy procedure by post prior to attending for the biopsy.

## Authors' contributions

DG, AV, MIM, PD, ETD, MW, VB, and TDS conceived and designed the experiments. AV, MND, AR, LP, DK, and AAB analyzed the data. DG, AKH, KSS, AB, EG, ACN, RD, PD, and FN contributed reagents, materials and analysis tools. DG, AV, MND, AR, MW, VB, and TD wrote the manuscript.

## Supplementary Material

Additional file 1 **Table S1:**Age for individuals with microarray expression in skin, adipose tissue, and LCLs. Click here for file

Additional file 2 **Table S2: ***P*values for ageeffect in skin tissue. Click here for file

Additional file 3 **Table S3: ***P*values for ageeffect in adipose tissue. Click here for file

Additional file 4 **Table S4:**List of genes with age effect in skin and adipose tissue included in the GenAge database [6]. Click here for file

Additional file 5 **Table S5: ***P*values for ageeffect in LCLs. Click here for file

Additional file 6 **Table S6: ***P*values for ageeffect in fresh lymphocytes. Click here for file

Additional file 7 **Figure S1:***P*values distribution for age effect analysis in 777 individuals with lymphocytes cell lines (LCLs) and 92 individuals with fresh lymphocytes expression arrays. Click here for file

Additional file 8 **Figure S2**: *P*values distribution for age effect analysis in 40 individuals with both lymphocytes cell lines (LCLs) and fresh lymphocytes expression profiles. Click here for file

Additional file 9 **Figure S3**: Beta values from lymphocytes cell lines (LCLs) and fresh lymphocytes expression association with age in 40 common samples (left) and in whole dataset (777 LCL and 92 fresh lymphocytes) (right). Click here for file

Additional file 10 **Table S7: ***P*values for ageeffect in brain. Click here for file

Additional file 11 **Table S8: ***P*values for genotype-by-age interactions in skin tissue. Click here for file

Additional file 12 **Table S9: ***P*values for genotype-by-age interactions in adipose tissue. Click here for file

Additional file 13 TableS10**: **List of contributors to the MuTHER and the UK Brain Expression Consortiums. Click here for file

## References

[B1] Reznick DN The genetic basis of aging: an evolutionary biologist's perspective. Sci Aging Knowl Environ 2005 14 pe7 10.1126/sageke.2005.11.pe7 15772417

[B2] Trabzuni D Wray S Vandrovcova J Ramasamy A Walker R Smith C Luk C Gibbs JR Dillman A Hernandez DG Arepalli S Singleton AB Cookson MR Pittman AM de Silva R Weale ME Hardy J Ryten M MAPT expression and splicing is differentially regulated by brain region: relation to genotype and implication for tauopathies. Hum Mol Genet 2012 14 4094 4103 10.1093/hmg/dds238 22723018 PMC3428157

[B3] Weindruch R Kayo T Lee C-K Prolla TA Gene expression profiling of aging using DNA microarrays. Mech Ageing Dev 2002 14 177 193 10.1016/S0047-6374(01)00344-X 11718811

[B4] Ly DH Lockhart DJ Lerner RA Schultz PG Mitotic misregulation and human aging. Science 2000 14 2486 2492 10.1126/science.287.5462.2486 10741968

[B5] Lu T Pan Y Kao S-Y Li C Kohane I Chan J Yankner BA Gene regulation and DNA damage in the ageing human brain. Nature 2004 14 883 891 10.1038/nature02661 15190254

[B6] Rodwell GEJ Sonu R Zahn JM Lund J Wilhelmy J Wang L Xiao W Mindrinos M Crane E Segal E Myers BD Brooks JD Davis RW Higgins J Owen AB Kim SK A transcriptional profile of aging in the human kidney. PLoS Biol 2004 14 e427 10.1371/journal.pbio.0020427 15562319 PMC532391

[B7] de Magalhaes JP Curado J Church GM Meta-analysis of age-related gene expression profiles identifies common signatures of aging. Bioinformatics 2009 14 875 881 10.1093/bioinformatics/btp073 19189975 PMC2732303

[B8] Grundberg E Small KS Hedman AK Nica AC Buil A Keildson S Bell JT Yang TP Meduri E Barrett A Nisbett J Sekowska M Wilk A Shin SY Glass D Travers M Min JL Ring S Ho K Thorleifsson G Kong A Thorsteindottir U Ainali C Dimas AS Hassanali N Ingle C Knowles D Krestyaninova M Lowe CE Di Meglio P Mapping cis- and trans-regulatory effects across multiple tissues in twins. Nat Genet 2012 14 1084 1089 10.1038/ng.2394 22941192 PMC3784328

[B9] Nica AC Parts L Glass D Nisbet J Barrett A Sekowska M Travers M Potter S Grundberg E Small K Hedman ÅK Bataille V Tzenova Bell J Surdulescu G Dimas AS Ingle C Nestle FO di Meglio P Min JL Wilk A Hammond CJ Hassanali N Yang T-P Montgomery SB O'Rahilly S Lindgren CM Zondervan KT Soranzo N Barroso I Durbin R The architecture of gene regulatory variation across multiple human tissues: The MuTHER Study. PLoS Genet 2011 14 e1002003 10.1371/journal.pgen.1002003 21304890 PMC3033383

[B10] Moayyeri A Hammond CJ Valdes AM Spector TD Cohort Profile: TwinsUK and healthy ageing twin study. Int J Epidemiol 2013 14 76 85 10.1093/ije/dyr207 22253318 PMC3600616

[B11] Huang DW Sherman BT Lempicki RA Systematic and integrative analysis of large gene lists using DAVID bioinformatics resources. Nat Protocols 2008 14 44 57 10.1038/nprot.2008.211 19131956

[B12] Sullivan PF Fan C Perou CM Evaluating the comparability of gene expression in blood and brain. Am J Med GenetB Neuropsychiatr Genet 2006 14 261 268 10.1002/ajmg.b.30272 16526044

[B13] Aguilera G HPA axis responsiveness to stress: Implications for healthy aging. Exp Gerontol 2011 14 90 95 10.1016/j.exger.2010.08.023 20833240 PMC3026863

[B14] Tacutu R Craig T Budovsky A Wuttke D Lehmann G Taranukha D Costa J Fraifeld VE de Magalhães JP Human Ageing Genomic Resources: Integrated databases and tools for the biology and genetics of ageing. Nucleic Acids Res 2013 14 D1027 D1033 10.1093/nar/gks1155 23193293 PMC3531213

[B15] Joehanes R Johnson AD Barb JJ Raghavachari N Liu P Woodhouse KA O'Donnell CJ Munson PJ Levy D Gene expression analysis of whole blood, peripheral blood mononuclear cells, and lymphoblastoid cell lines from the Framingham Heart Study. Physiol Genomics 2012 14 59 75 10.1152/physiolgenomics.00130.2011 22045913 PMC3289123

[B16] Trabzuni D Ryten M Walker R Smith C Imran S Ramasamy A Weale ME Hardy J Quality control parameters on a large dataset of regionally dissected human control brains for whole genome expression studies. J Neurochem 2011 14 275 282 10.1111/j.1471-4159.2011.07432.x 21848658 PMC3664422

[B17] Insinga A Monestiroli S Ronzoni S Carbone R Pearson M Pruneri G Viale G Appella E Pelicci P Minucci S Impairment of p53 acetylation, stability and function by an oncogenic transcription factor. Embo J 2004 14 1144 1154 10.1038/sj.emboj.7600109 14976551 PMC380970

[B18] Menschikowski M Hagelgans A Tiebel O Klinsmann L Eisenhofer G Siegert G Expression and shedding of endothelial protein C receptor in prostate cancer cells. Cancer Cell Int 2011 14 4 10.1186/1475-2867-11-4 21320357 PMC3045874

[B19] Proescholdt MA Mayer C Kubitza M Schubert T Liao S-Y Stanbridge EJ Ivanov S Oldfield EH Brawanski A Merrill MJ Expression of hypoxia-inducible carbonic anhydrases in brain tumors. NeuroOncol 2005 14 465 475 10.1215/S1152851705000025 PMC1871734 16212811

[B20] Ohsawa S Miura M Caspase-mediated changes in Sir2α during apoptosis. FEBS Letters 2006 14 5875 5879 10.1016/j.febslet.2006.09.051 17027980

[B21] Luo J Nikolaev AY Imai S-i Chen D Su F Shiloh A Guarente L Gu W Negative control of p53 by Sir2α promotes cell survival under stress. Cell 2001 14 137 148 10.1016/S0092-8674(01)00524-4 11672522

[B22] Brunet A Sweeney LB Sturgill JF Chua KF Greer PL Lin Y Tran H Ross SE Mostoslavsky R Cohen HY Hu LS Cheng H-L Jedrychowski MP Gygi SP Sinclair DA Alt FW Greenberg ME Stress-dependent regulation of FOXO transcription factors by the SIRT1 deacetylase. Science 2004 14 2011 2015 10.1126/science.1094637 14976264

[B23] Sakata T Ferdous G Tsuruta T Satoh T Baba S Muto T Ueno A Kanai Y Endou H Okayasu I L-type amino-acid transporter 1 as a novel biomarker for high-grade malignancy in prostate cancer. Pathol Int 2009 14 7 18 10.1111/j.1440-1827.2008.02319.x 19121087

[B24] Hollingworth P Harold D Sims R Gerrish A Lambert J-C Carrasquillo MM Abraham R Hamshere ML Pahwa JS Moskvina V Dowzell K Jones N Stretton A Thomas C Richards A Ivanov D Widdowson C Chapman J Lovestone S Powell J Proitsi P Lupton MK Brayne C Rubinsztein DC Gill M Lawlor B Lynch A Brown KS Passmore PA Craig D Common variants at ABCA7, MS4A6A/MS4A4E, EPHA1, CD33 and CD2AP are associated with Alzheimer's disease. Nat Genet 2011 14 429 435 10.1038/ng.803 21460840 PMC3084173

[B25] Misra V Lee H Singh A Huang K Thimmulappa RK Mitzner W Biswal S Tankersley CG Global expression profiles from C57BL/6J and DBA/2J mouse lungs to determine aging-related genes. Physiol Genomics 2007 14 429 440 10.1152/physiolgenomics.00060.2007 17726092

[B26] Grundberg E Kwan T Ge B Lam KCL Koka V Kindmark A Mallmin H Dias J Verlaan DJ Ouimet M Sinnett D Rivadeneira F Estrada K Hofman A van Meurs JM Uitterlinden A Beaulieu P Graziani A Harmsen E Ljunggren Ö Ohlsson C Mellström D Karlsson MK Nilsson O Pastinen T Population genomics in a disease targeted primary cell model. Genome Res 2009 14 1942 1952 10.1101/gr.095224.109 19654370 PMC2775606

[B27] Grundberg E Small KS Hedman AK Nica AC Buil A Keildson S Bell JT Yang T-P Meduri E Barrett A Nisbett J Sekowska M Wilk A Shin S-Y Glass D Travers M Min JL Ring S Ho K Thorleifsson G Kong A Thorsteindottir U Ainali C Dimas AS Hassanali N Ingle C Knowles D Krestyaninova M Lowe CE Di Meglio P Mapping cis- and trans-regulatory effects across multiple tissues in twins. Nat Genet 2012 14 1084 1089 10.1038/ng.2394 22941192 PMC3784328

[B28] Njajou OT Blackburn EH Pawlikowska L Mangino M Damcott CM Kwok P-Y Spector TD Newman AB Harris TB Cummings SR Cawthon RM Shuldiner AR Valdes AM Hsueh W-C A common variant in the telomerase RNA component is associated with short telomere length. PLoS ONE 2010 14 e13048 10.1371/journal.pone.0013048 20885959 PMC2946401

[B29] McKay JD Hung RJ Gaborieau V Boffetta P Chabrier A Byrnes G Zaridze D Mukeria A Szeszenia-Dabrowska N Lissowska J Rudnai P Fabianova E Mates D Bencko V Foretova L Janout V McLaughlin J Shepherd F Montpetit A Narod S Krokan HE Skorpen F Elvestad MB Vatten L Njolstad I Axelsson T Chen C Goodman G Barnett M Loomis MM Lung cancer susceptibility locus at 5p15.33. Nat Genet 2008 14 1404 1406 10.1038/ng.254 18978790 PMC2748187

[B30] Martin GM Austad SN Johnson TE Genetic analysis of ageing: role of oxidative damage and environmental stresses. Nat Genet 1996 14 25 34 10.1038/ng0596-25 8673100

[B31] Wheeler HE Kim SK Genetics and genomics of human ageing. Philos Trans R SocLond B Biol Sci 2011 14 43 50 10.1098/rstb.2010.0259 PMC3001305 21115529

[B32] Zahn JM Sonu R Vogel H Crane E Mazan-Mamczarz K Rabkin R Davis RW Becker KG Owen AB Kim SK Transcriptional profiling of aging in human muscle reveals a common aging signature. PLoS Genet 2006 14 e115 10.1371/journal.pgen.0020115 16789832 PMC1513263

[B33] Forte E Luftig MA MDM2-dependent inhibition of p53 is required for Epstein-Barr virus B-cell growth transformation and infected-cell survival. J Virol 2009 14 2491 2499 10.1128/JVI.01681-08 19144715 PMC2648290

[B34] Viñuela A Snoek LB Riksen JAG Kammenga JE Genome-wide gene expression regulation as a function of genotype and age in C. elegans. Genome Res 2010 14 929 937 10.1101/gr.102160.109 20488933 PMC2892094

[B35] Viñuela A Snoek LB Riksen JAG Kammenga JE Aging uncouples heritability and expression-QTL in Caenorhabditis elegans. G3 (Bethesda) 2012 14 597 605 2012 22670229 10.1534/g3.112.002212 PMC3362942

[B36] Wheeler HE Metter EJ Tanaka T Absher D Higgins J Zahn JM Wilhelmy J Davis RW Singleton A Myers RM Ferrucci L Kim SK Sequential use of transcriptional profiling, expression quantitative trait mapping, and gene association implicates MMP20 in human kidney aging. PLoS Genet 2009 14 e1000685 10.1371/journal.pgen.1000685 19834535 PMC2752811

[B37] Kent JW Jr Göring HHH Charlesworth JC Drigalenko E Diego VP Curran JE Johnson MP Dyer TD Cole SA Jowett JBM Mahaney MC Comuzzie AG Almasy L Moses EK Blangero J Williams-Blangero S Genotype × age interaction in human transcriptional ageing. Mech Ageing Dev 2012 14 581 590 10.1016/j.mad.2012.07.005 22871458 PMC3541784

[B38] Herndon LA Schmeissner PJ Dudaronek JM Brown PA Listner KM Sakano Y Paupard MC Hall DH Driscoll M Stochastic and genetic factors influence tissue-specific decline in ageing C. elegans. Nature 2002 14 808 814 10.1038/nature01135 12397350

[B39] Law MH Montgomery GW Brown KM Martin NG Mann GJ Hayward NK MacGregor S Meta-analysis combining new and existing data sets confirms that the TERT-CLPTM1L locus influences melanoma risk. J Invest Dermatol 2012 14 485 487 10.1038/jid.2011.322 21993562 PMC3258346

[B40] Janzen V Forkert R Fleming HE Saito Y Waring MT Dombkowski DM Cheng T DePinho RA Sharpless NE Scadden DT Stem-cell ageing modified by the cyclin-dependent kinase inhibitor p16INK4a. Nature 2006 14 421 426 16957735 10.1038/nature05159

[B41] Acosta JC O'Loghlen A Banito A Guijarro MV Augert A Raguz S Fumagalli M Da Costa M Brown C Popov N Takatsu Y Melamed J d'Adda di Fagagna F Bernard D Hernando E Gil J Chemokine signaling via the CXCR2 receptor reinforces senescence. Cell 2008 14 1006 1018 10.1016/j.cell.2008.03.038 18555777

[B42] Michaloglou C Vredeveld LCW Mooi WJ Peeper DS BRAFE600 in benign and malignant human tumours. Oncogene 2007 14 877 895 17724477 10.1038/sj.onc.1210704

[B43] Serrano M Blasco MA Cancer and ageing: convergent and divergent mechanisms. Nat Rev Mol Cell Biol 2007 14 715 722 10.1038/nrm2242 17717516

[B44] Acosta JC Gil J Senescence: a new weapon for cancer therapy. Trends Cell Biol 2012 14 211 219 10.1016/j.tcb.2011.11.006 22245068

[B45] Maser RS DePinho RA Connecting chromosomes, crisis, and cancer. Science 2002 14 565 569 10.1126/science.297.5581.565 12142527

[B46] Maser RS DePinho RA Keeping telomerase in its place. Nat Med 2002 14 934 936 10.1038/nm0902-934 12205452

[B47] O'Hagan RC Chang S Maser RS Mohan R Artandi SE Chin L DePinho RA Telomere dysfunction provokes regional amplification and deletion in cancer genomes. Cancer Cell 2002 14 149 155 10.1016/S1535-6108(02)00094-6 12204535

[B48] Meeker AK Hicks JL Gabrielson E Strauss WM De Marzo AM Argani P Telomere shortening occurs in subsets of normal breast epithelium as well as in situ and invasive carcinoma. AmJPathol 2004 14 925 935 10.1016/S0002-9440(10)63180-X PMC1614707 14982846

[B49] Choudhury AR Ju Z Djojosubroto MW Schienke A Lechel A Schaetzlein S Jiang H Stepczynska A Wang C Buer J Lee H-W von Zglinicki T Ganser A Schirmacher P Nakauchi H Rudolph KL Cdkn1a deletion improves stem cell function and lifespan of mice with dysfunctional telomeres without accelerating cancer formation. Nat Genet 2007 14 99 105 10.1038/ng1937 17143283

[B50] Feng Z Levine AJ The regulation of energy metabolism and the IGF-1/mTOR pathways by the p53 protein. Trends Cell Biol 2010 14 427 434 10.1016/j.tcb.2010.03.004 20399660 PMC2921989

[B51] Feng Z Hu W de Stanchina E Teresky AK Jin S Lowe S Levine AJ The regulation of AMPK β1, TSC2, and PTEN expression by p53: stress, cell and tissue specificity, and the role of these gene products in modulating the IGF-1-AKT-mTOR pathways. Cancer Res 2007 14 3043 3053 10.1158/0008-5472.CAN-06-4149 17409411

[B52] The MuTHER project (Multiple Tissue Human Expression Resource). http://www.muther.ac.uk

[B53] The R Project for Statistical Computing. http://www.r-project.org/

[B54] Bates D Maechler M Bolker B lme4: Linear mixed-effects models using S4 classes. Rpackage version 0.999375-41. 2011

[B55] Dobbin K Shih JH Simon R Statistical design of reverse dye microarrays. Bioinformatics 2003 14 803 810 10.1093/bioinformatics/btg076 12724289

[B56] Millar T Walker R Arango JC Ironside JW Harrison DJ MacIntyre DJ Blackwood D Smith C Bell JE Tissue and organ donation for research in forensic pathology: the MRC Sudden Death Brain and Tissue Bank. J Pathol 2007 14 369 375 10.1002/path.2247 17990279

[B57] International Parkinson's Disease Genomics C Wellcome Trust Case Control C A two-stage meta-analysis identifies several new loci for Parkinson's disease. PLoS Genet 2011 14 e1002142 10.1371/journal.pgen.1002142 21738488 PMC3128098

[B58] International Parkinson Disease Genomics Consortium Nalls MA Plagnol V Hernandez DG Sharma M Sheerin UM Saad M Simon-Sanchez J Schulte C Lesage S Sveinbjornsdottir S Stefansson K Martinez M Hardy J Heutink P Brice A Gasser T Singleton AB Wood NW Imputation of sequence variants for identification of genetic risks for Parkinson's disease: a meta-analysis of genome-wide association studies. Lancet 2011 14 641 649 21292315 10.1016/S0140-6736(10)62345-8 PMC3696507

[B59] Li Y Willer C Sanna S Abecasis G Genotype imputation. Annu Rev Genomics Hum Genet 2009 14 387 406 10.1146/annurev.genom.9.081307.164242 19715440 PMC2925172

[B60] Li Y Willer CJ Ding J Scheet P Abecasis GR MaCH: using sequence and genotype data to estimate haplotypes and unobserved genotypes. Genet Epidemiol 2010 14 816 834 10.1002/gepi.20533 21058334 PMC3175618

[B61] Minimac. http://genome.sph.umich.edu/wiki/Minimac

[B62] The UK Brain Expression Consortium and expression resource.

