# Supplementary figures and images for: Gene expression changes with age in skin, adipose tissue, blood and brain
Source: Genome Biol. 2013 Jul 26;14(7):R75. doi: 10.1186/gb-2013-14-7-r75 (PMC4054017; doi:10.1186/gb-2013-14-7-r75)

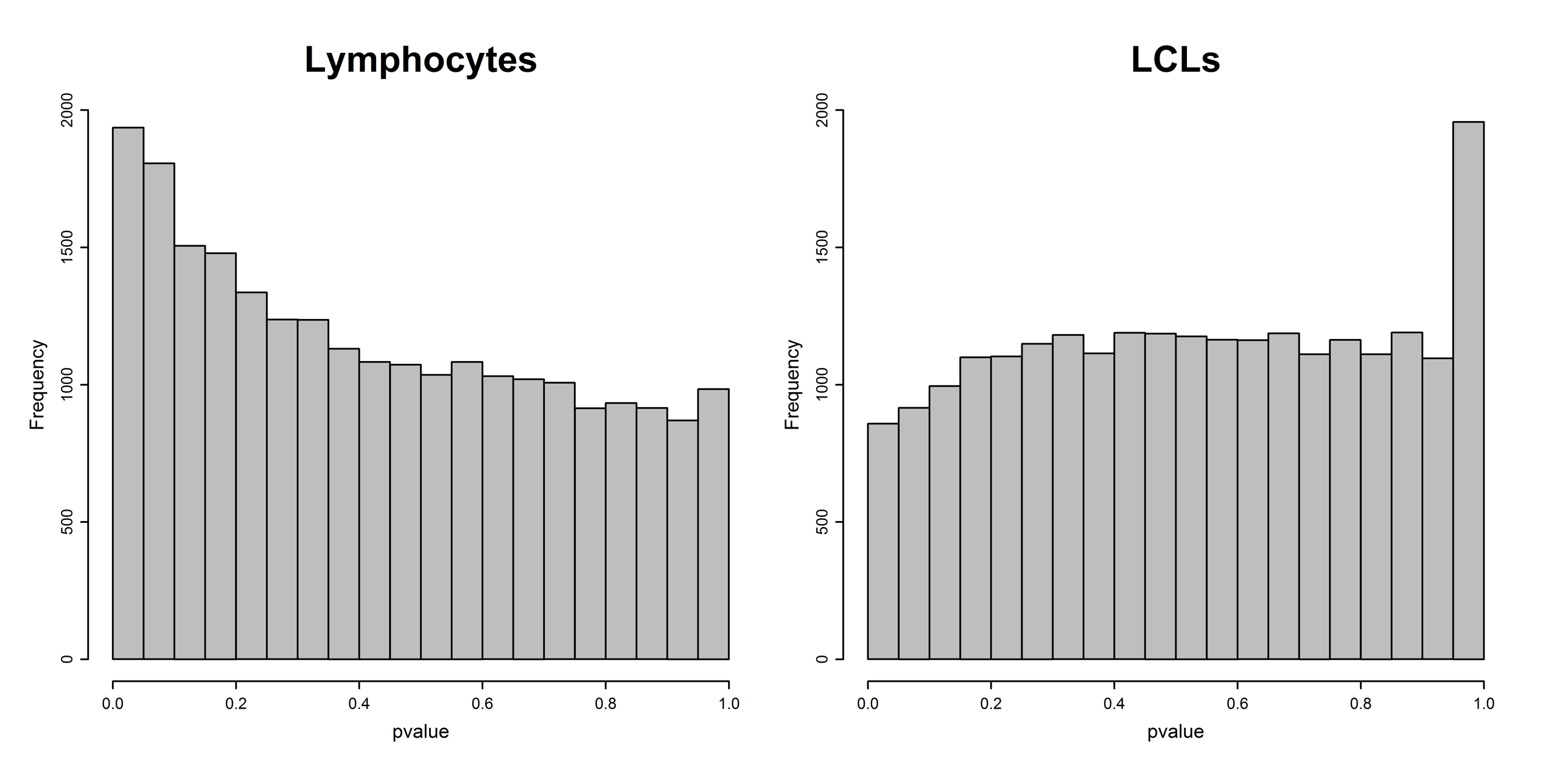

Supplement: Additional file 7 — Figure S1:Pvalues distribution for age effect analysis in 777 individuals with lymphocytes cell lines (LCLs) and 92 individuals with fresh lymphocytes expression arrays. [file gb-2013-14-7-r75-S7.TIFF]

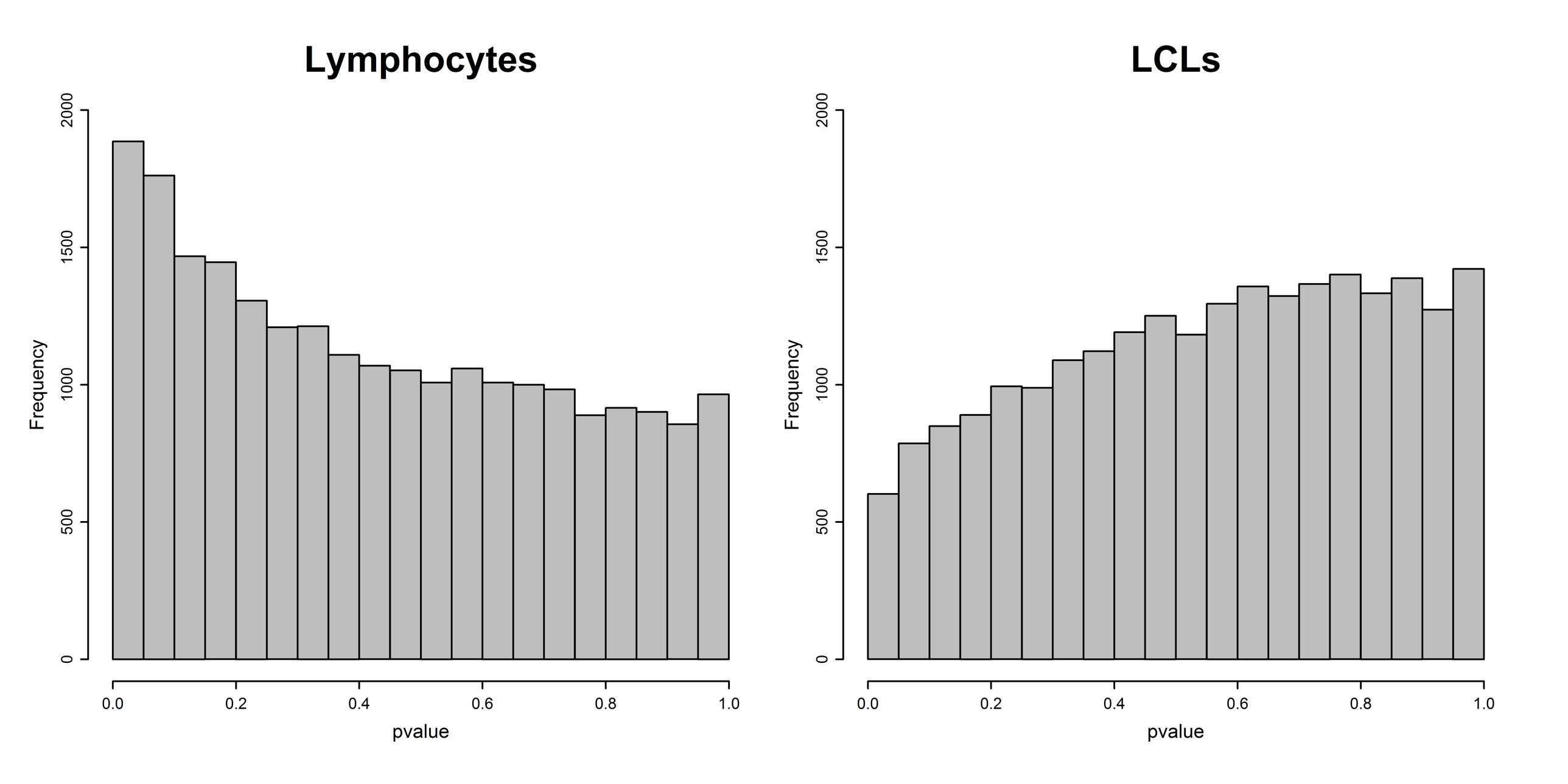

Supplement: Additional file 8 — Figure S2: Pvalues distribution for age effect analysis in 40 individuals with both lymphocytes cell lines (LCLs) and fresh lymphocytes expression profiles. [file gb-2013-14-7-r75-S8.TIFF]

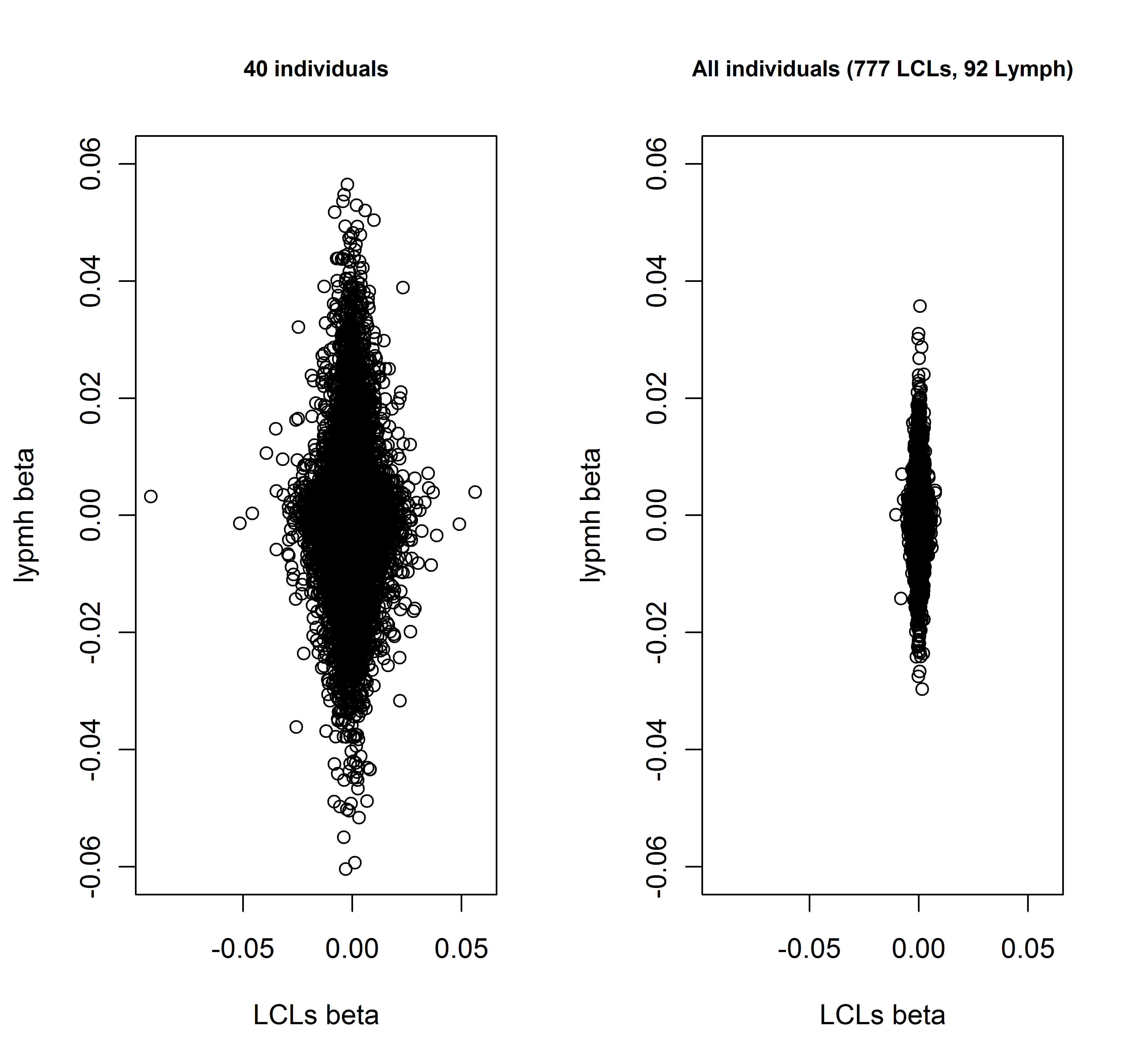

Supplement: Additional file 9 — Figure S3: Beta values from lymphocytes cell lines (LCLs) and fresh lymphocytes expression association with age in 40 common samples (left) and in whole dataset (777 LCL and 92 fresh lymphocytes) (right). [file gb-2013-14-7-r75-S9.TIFF]
