# Supplementary material for: Gene expression changes with age in skin, adipose tissue, blood and brain
Source: Genome Biol. 2013 Jul 26;14(7):R75. doi: 10.1186/gb-2013-14-7-r75 (PMC4054017; doi:10.1186/gb-2013-14-7-r75)
Supplement: Additional file 13 — TableS10: List of contributors to the MuTHER and the UK Brain Expression Consortiums. [file gb-2013-14-7-r75-S13.DOCX]

**The UK Human Brain Expression Consortium comprises:**

John A Hardy^1^, Mina Ryten^1^, Daniah Trabzuni^1^, Michael Weale^2^, Adaikalavan Ramasamy^2^, Colin Smith^3^ & Robert Walker^3^

1) Department of Molecular Neuroscience, UCL Institute of Nerurology, London, UK

2) Department of Medical & Molecular Genetics, King's College London, UK

3) Department of Pathology, The University of Edinburgh, Wilkie Building, Teviot Place, Edinburgh, UK

**Members of the MuTHER (Multiple Tissue Human Expression Resource) Consortium**

Kourosh R Ahmadi^1^, Chrysanthi Ainali^2^, Amy Barrett^3^, Veronique Bataille^1^, Jordana T Bell^1,4^, Alfonso Buil^5^, Panos Deloukas^6^, Emmanouil T Dermitzakis^5^, Antigone S Dimas^4,5^, Richard Durbin^6^, Daniel Glass^1^, Elin Grundberg^1,6^, Neelam Hassanali^3^, Åsa K Hedman^4^, Catherine Ingle^6^, David Knowles^7^, Maria Krestyaninova^8^, Cecilia M Lindgren^4^, Christopher E Lowe^9,10^, Mark I McCarthy^3,4,11^, Eshwar Meduri^1,6^, Paola di Meglio^12^, Josine L Min^4^, Stephen B Montgomery^5^, Frank O Nestle^12^, Alexandra C Nica^5^, James Nisbet^6^, Stephen O’Rahilly^9,10^, Leopold Parts^6^, Simon Potter^6^, Magdalena Sekowska^6^, So-Youn Shin^6^, Kerrin S Small^1, 6^, Nicole Soranzo^6, 1^, Tim D Spector^1^, Gabriela Surdulescu^1^, Mary E Travers^3^, Loukia Tsaprouni^6^, Sophia Tsoka^2^, Alicja Wilk^6^, Tsun-Po Yang^6^, Krina T. Zondervan^4^

**Affiliations**

1.Department of Twin Research and Genetic Epidemiology, King's College London, London, UK

2. Department of Informatics, School of Natural and Mathematical Sciences, King’s College London, Strand, London WC2R 2LS.

3. Oxford Centre for Diabetes, Endocrinology & Metabolism, University of Oxford, Churchill Hospital, Oxford, UK

4. Wellcome Trust Centre for Human Genetics, University of Oxford, Oxford, UK.

5. Department of Genetic Medicine and Development, University of Geneva Medical School, Geneva, Switzerland

6.Wellcome Trust Sanger Institute, Wellcome Trust Genome Campus, Hinxton, UK

7. University of Cambridge, Cambridge, UK

8. European Bioinformatics Institute, Hinxton, UK

9. University of Cambridge Metabolic Research Labs, Institute of Metabolic Science Addenbrooke’s Hospital Cambridge, UK

10. Cambridge NIHR Biomedical Research Centre, Addenbrooke’s Hospital, Cambridge, UK

11. Oxford NIHR Biomedical Research Centre, Churchill Hospital, Oxford, UK

12. St. John's Institute of Dermatology, King's College London, London, UK
